# Association between motoric cognitive risk syndrome and future falls among Chinese community‐dwelling elderly: A nationwide cohort study

**DOI:** 10.1002/brb3.3044

**Published:** 2023-05-18

**Authors:** Wei‐wei Lu, Bang‐zhong Liu, Min‐zhi Lv, Jin‐kang Tu, Zi‐rui Kang, Rui‐Ping Hu, Yu‐Lian Zhu, Jian Zhang

**Affiliations:** ^1^ Department of Rehabilitation Medicine Shanghai Geriatric Medical Center Shanghai China; ^2^ Department of Rehabilitation Medicine, Zhongshan Hospital Fudan University Shanghai China; ^3^ Department of Medical Statistics, Zhongshan Hospital Fudan University Shanghai China; ^4^ Department of Rehabilitation Medicine, Huashan Hospital Fudan University Shanghai China

**Keywords:** elderly, falls, memory, walking speed

## Abstract

**Background:**

Motoric Cognitive Risk syndrome (MCR), known as the predementia stage, is characterized by both subjective cognitive complaint (SCC) and slow gait. This study aimed to investigate the causal relationship between MCR, its components, and falls.

**Methods:**

Participants aged ≥ 60 years were selected from China Health and Retirement Longitudinal Study. SCC was determined by participants' responses to the question “How would you rate your memory at present?” with “poor” being the indicative answer. Slow gait was defined as one standard deviation or more below age‐ and gender‐appropriate mean values of gait speed. MCR was identified when both SCC and slow gait were presented. Future falls were investigated by the question “have you fallen down during follow‐up until wave 4 in 2018?” Logistic regression analysis was performed to test the longitudinal association of MCR, its components and future falls during the following 3 years.

**Results:**

Of 3748 samples in this study, the prevalence of MCR, SCC, and slow gait was 5.92%, 33.06%, and 15.21%, respectively. MCR increased the risk of falls during the following 3 years by 66.7% compared to non‐MCR after controlling for covariates. In the fully adjusted models, with the healthy group as reference, MCR (*OR* = 1.519, 95%CI = 1.086–2.126) and SCC (*OR* = 1.241, 95%CI = 1.018–1.513), but not slow gait, increased the risk of future falls.

**Conclusions:**

MCR independently predicts future falls risk in the following 3 years. Measuring MCR can be a pragmatic tool for early identification of falls risk.

## Graphical abstract

The association of motoric cognitive risk syndrome (MCR) with falls was analyzed using the logistic regression model. MCR increased the risk of future falls during the following 3 years by 41.6% in the Chinese elderly cohort in the fully adjusted model.

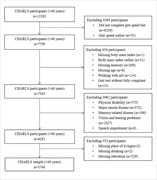

## 1 INTRODUCTION

Falls and fall‐related injuries, which lead to morbidity and mortality, are on the rise among the elderly aged 60 and above (Hopewell et al., 2018; Khow & Visvanathan, 2017). Falls are often accidental incidences accompanied by cumulative risks associated with intrinsic (including poor muscle strength of lower limb, balance problems, and dementia) and extrinsic factors (including poor lighting or chaos at the place of living) (Beauchet et al., 2011; Cuevas‐Trisan, 2017). Although there are currently up to 26 screening tools for fall risks, these tools still lack higher predictive validity for detecting the degree of fall risk (Park, 2018). Thus, simple and practical assessment tools for assessing cumulative fall risk are essential for fall prevention.

Over the past decade, motoric cognitive risk syndrome (MCR) has been known as a predementia syndrome without major neurocognitive impairment in cognitively normal individuals (Verghese et al., 2013). MCR combines two clinical characteristics: subjective cognitive complaint (SCC) and slow gait (Verghese et al., 2014a, 2014b). The significance of MCR as an independent risk of dementia has been confirmed in different populations, but MCR as a screening tool for falls still needs more exploration in different countries. Callisaya et al. (2016) conducted an investigation into the correlation between MCR and falls in the population aged ≥60 years across five cohort studies in three different countries. This study revealed a significant association in only three of the cohorts, while no association was found in the other two (Callisaya et al., 2016). Similar results were also observed in slow gait and SCC (Callisaya et al., 2016). Discrepant results regarding the association between MCR and falls risk have been observed in various cohort studies. For instance, Beauchet et al. (2019) found that MCR could predict future falls risk in French female adults, whereas Lord et al. (2020) reported no such association in one of the two ethnic groups in New Zealand. These inconsistent findings may be attributed to variations in factors such as race, age, gender, and other potential factors among research participants. Hence, the findings of existing studies may not necessarily apply to other races, countries, and age groups. Additionally, the samples in some studies were collected around two decades ago (Beauchet et al., 2019), indicating that the association between MCR and falls risk needs to be updated.

Furthermore, the association of SCC and slow gait with falls still needs to be confirmed, since the related findings were opposite in some studies (Beauchet et al., 2019; Yuan et al., 2021). Slow gait was found to be statistically associated with falls in patients with cognitive impairment (MacAulay et al., 2021; Pieruccini‐Faria et al., 2020). For the individuals with mild cognitive impairment (MCI), the risk of falls was significantly increased during the transition from fast‐to‐slow walking speed (Boripuntakul et al., 2022). However, no association of slow gait and falls was observed in some cohorts (Callisaya et al., 2016). SCC was considered as an earlier sign of cognitive decline which could impact gait characteristics and balance, ending with an increase in the falls risk (O'Keefe et al., 2018). SCC also affected the ability to process and respond to the surroundings, which increased the falls risk.

Based on these inconsistent existing findings, a prospective longitudinal study using a large sample is needed to test the causal relationship between MCR, its components and falls. We hypothesized that MCR and its components (SCC and slow gait) could separately predict risk of falls. To test this hypothesis, we conducted a cohort study on the community‐dwelling elderly recruited from the China Health and Retirement Longitudinal Study (CHARLS) (Zhao et al., 2014).

## 2 METHODS

### 2.1 Study participants

**FIGURE 1:**
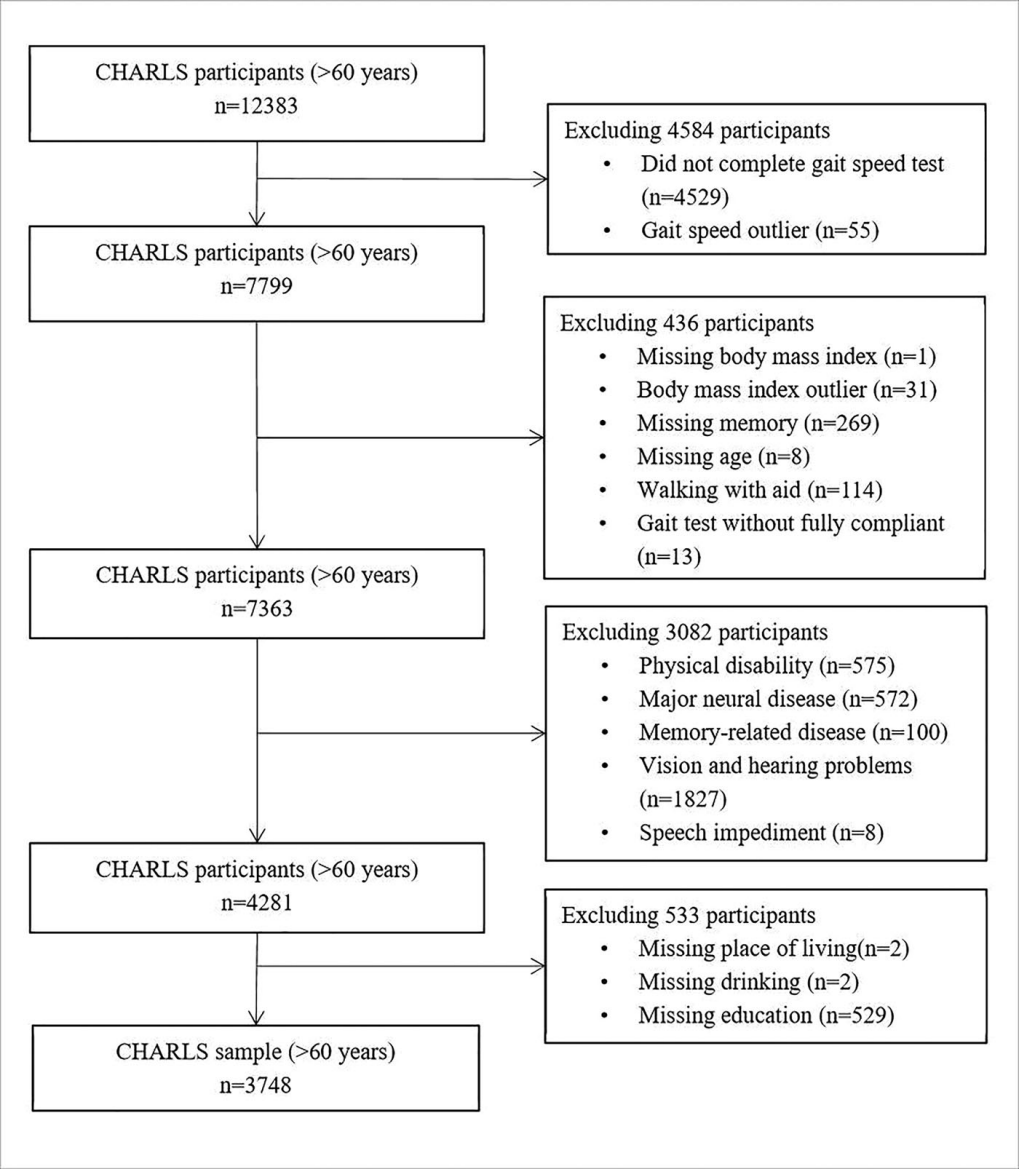
Flowchart of participants’ selection. A sample of 3748 participants was included in this study. CHARLS, the China Health and Retirement Longitudinal Study.

We utilized the national baseline data from CHARLS wave 3, which was collected in the year 2015, along with data of falls history from wave 2 (collected in 2013) and wave 3, and data of future falls during the follow‐up from wave 4 (collected in 2018). CHARLS is an observational cohort study, hosted by Peking University, designed to analyze health and aging‐related issues among middle‐aged and older Chinese. The study commenced in June 2011 and has been collecting data every 2 years, including residents aged 45 years and older in 450 counties or villages. The CHARLS data are freely available on the China Health and Retirement Longitudinal Study website for nonprofit use. The inclusion criteria for our study were individuals who were 60 years older or above, completed the CHARLS questionnaire independently, were able to ambulate, and had complete data on all variables. The exclusion criteria were physical disabilities (including limb motor dysfunction), major neural disease (including stroke, brain damage, and mental retardation), memory‐related disease (including Parkinson's disease, dementia, and brain atrophy), vision and hearing problems, and speech impediments. The final sample comprised 3748 participants (Figure 1).

### 2.2 Definition of MCR

Recently, MCR has been considered as a predementia stage, characterized by the presence of SCC and slow gait, without major neurocognitive or mobility disorders (Verghese et al., 2014a, 2013). The assessment of SCC involved asking participants to rate their memory at present, using the question “how would you rate your memory at present? Would you say it is excellent, very good, good, fair, or poor?” Participants who rated their self‐memory as “poor” were considered as having SCC. Walking speed was measured by asking participants to walk straight at their usual pace over a distance of 2.5 m in a flattened area. If the participants recently had surgery, trauma, or had other health dysfunctions, they did not undergo the walking speed test. Walking speed was calculated as the average of two trials in meters per second. The mean and SD of walking speed were calculated by both gender and age (e.g., female aged 60 to 65 years old). The cutoff speed for slow gait was defined as one standard deviation (SD) or more below age‐ and gender‐appropriate mean values established in the present cohort (Verghese et al., 2014a). The data on both SCC and gait speed were collected face‐to‐face in the year 2015, wave 3 of CHARLS.

### 2.3 Definition of future falls

An international consensus definition of a fall was described as an unexpected incident in which individuals come to rest or slip on a lower level or floor (Lamb et al., 2005). For the purposes of this study, future falls were identified by the question, “have you fallen down during follow‐up until wave 4 in 2018?” The way of measuring falls in this study was similar to the Health and Retirement Study (HRS) conducted in the United States.

### 2.4 Measurements of covariates

In this study, demographic characteristics, behavioral habits, falls history and chronic diseases were included as the covariates, which were collected in CHARLS wave 3. Demographic characteristics comprised age (in years), gender, level of education, body mass index (BMI) calculated as weight divided by height (kg/m^2^), place of living, and cohabitation status. Behavioral habits consisted of alcohol consumption and smoking. Falls history was defined as “have you fallen down before wave 3?” Chronic diseases were self‐reported physician‐diagnosed, including emotional and psychiatric problems, hypertension, diabetes, chronic lung disease, arthritis (including rheumatic arthritis), and heart disease (such as heart attack, angina, coronary heart disease, or other heart problems). The level of education was re‐defined into 3 groups in CHARLS: primary school and below, middle school, and completion of at least the first year in college or university. Place of living was categorized into city zone and other areas (including villages, the town center, and combination zone between urban and rural areas). Cohabitation status indicated whether or not the participants were living alone. Alcohol consumption was defined as consuming all types of liquor, beer, and wine at least once a week (Ma et al., 2020). Smoking was categorized into nonsmoking (including ex‐smoking) and current smoking. Besides physician‐diagnosed or self‐reported chronic diseases, the confirmation of medication and therapy for these diseases was also included in the definition of chronic diseases.

### 2.5 Standard ethical approvals and patient consent

The CHARLS was conducted by the principles of the Declaration of Helsinki. The ethical approval was received from Peking University's institutional review board (IRB00001052‐11015). The interviewees’ informed written consent was obtained before participating in CHARLS.

### 2.6 Statistics

The baseline characteristics of the participants were summarized by SSC, slow gait, and MCR using descriptive statistics, which reported mean and standard deviation (SD) for continuous variables with a normal distribution, or median and interquartile ranges for continuous variables with a nonnormal distribution, and frequencies and percentages for categorical variates. Chi‐square tests for categorical variables and Wilcoxon rank‐sum tests for continuous variables were used to examine differences in the characteristics of participants with and without MCR. The primary outcome of the association between future falls and MCR was tested using logistic regression models. Model 1 included MCR as the independent variable and falls as the dependent variable. Model 2 included demographic characteristics in addition to variables in Model 1. Model 3 included behavioral habits in addition to variables in Model 2. Model 4 included falls history and chronic diseases in addition to variables in Model 3. Further analysis was explored by dividing the participants into four groups: the MCR group, only SCC group, only slow gait group, and the healthy group. The association of future falls with MCR, SCC, and slow gait was tested by logistic regression analysis with adjusted covariates. Sensitivity analysis was performed by using univariate logistic regression after excluding participants with falls history. Odds ratio (OR) and 95% confidence interval (CI) were reported for regression results. All reported *p* values were two‐tailed with a significance level of .05. All statistical analyses were performed using STATA software (version 16.0; Stata Corp LP. TX).

## 3 RESULTS

### 3.1 Participant demographics

Table 1 presented the characteristics of the participants, with 1239 (33.06%) having SCC, 570 (15.21%) with slow gait, and 222 (5.92%) with MCR. Generally, of the participants, 18.36% (*n* = 688) reported falls during the follow‐up, and 25.35% (*n* = 950) reported fall history before CHARLS wave 3. The mean age of the participants was 67.211 (SD: 6.037) years, with 1843 participants (49.17%) being male and 2872 participants (76.63%) having complete primary school or below. The participants with MCR were more likely to be women, with primary education or below, and residing in the village or rural areas.

**TABLE 1:** Participant characteristics by SCC, slow gait, and MCR (*n* = 3748).

*n*	Total (*n* = 3748 [100%])	SCC (*n* = 1239 [33.06%])	Slow gait (*n* = 570 [15.21%])	MCR (*n* = 222 [5.92%])	*p* (MCR & non‐MCR)
Age (SD)	67.21 ± 6.04	67.43 ± 6.22	67.35 ± 6.04	67.17 ± 5.90	.987
Gender					.001^**^
Male (%)	1843 (49.17)	457 (36.88)	280 (49.12)	85 (38.29)	
Female (%)	1905 (50.83)	782 (63.12)	290 (50.88)	137 (61.71)	
Education level (%)					<.001^***^
≤Primary school	2872 (76.63)	1086 (87.65)	489 (85.79)	204 (91.89)	
Middle school	715 (19.08)	134 (10.82)	68 (11.93)	15 (6.76)	
≥High school	161 (4.30)	19 (1.53)	13 (2.28)	3 (1.35)	
BMI (SD)	23.48 ± 3.64	23.33 ± 3.54	23.49 ± 4.01	23.34 ± 3.98	.280
Place of living					<.001^***^
City residents (%)	894 (23.85)	188 (15.17)	109 (19.12)	23 (10.36)	
Others	2854 (76.15)	1051 (84.83)	461 (80.88)	199 (89.64)	
Cohabitation status					.660
Cohabitated (%)	3117 (83.16)	1024 (82.65)	464 (81.4)	187 (84.23)	
Others (%)	631 (16.84)	215 (17.35)	106 (18.6)	35 (15.77)	
Current drinkers (%)	1270 (33.88)	256 (20.66)	173 (30.35)	53 (23.87)	.001^**^
Current smokers (%)	1034 (27.59)	256 (20.66)	147 (25.79)	43 (19.37)	.005^**^
Hypertension (%)	1338 (35.70)	480 (38.74)	222 (38.95)	93 (41.89)	.047^*^
Diabetes (%)	328 (8.75)	112 (9.04)	53 (9.30)	23 (10.36)	.382
EPP (%)	44 (1.17)	18 (1.45)	11 (1.93)	6 (2.70)	.029^*^
CLD (%)	480 (12.81)	161 (12.99)	89 (15.61)	31 (13.96)	.595
Arthritis (%)	1423 (37.97)	575 (46.41)	238 (41.75)	101 (45.50)	.017^*^
Heart disease (%)	545 (14.54)	188 (15.17)	90 (15.79)	30 (13.51)	.654
Future falls (%)	688 (18.36)	293 (23.65)	114 (20.00)	59 (26.58)	.001^**^
Falls history (%)	950 (25.35)	390 (31.48)	170 (29.82)	71 (31.98)	.019^*^

Abbreviations: BMI, body mass index; CLD, chronic lung disease; EPP, emotional and psychiatric problems; MCR, motoric cognitive risk syndrome; non‐MCR, participants without SCC or slow gait; SCC, subjective cognitive complaint; SD, standard deviation.

*p < .05, **p < .01, ***p < .001.

### 3.2 MCR associated with future falls during follow‐up

In the univariate logistic model examining MCR as a predictor of falls, MCR was associated with a 66.7% increase in falls during 3 years of follow‐up compared to those without MCR (Table 2; Model 1). After controlling for covariates in Models 2, 3, and 4, the association of MCR with falls slightly attenuated but remained significant (OR = 1.507, 95%CI = 1.100–2.065; OR = 1.513, 95%CI = 1.105–2.074; OR = 1.416, 95%CI = 1.023–1.959) (Table 2; All variables used in the models displayed in eTable S1).

**TABLE 2:** Logistic regression analysis of future falls (dependent variable) and MCR (independent variables) (*n* = 3748).

Variable	Model 1^a^	Model 2^b^	Model 3^c^	Model 4^d^
	*OR* [95%CI]	*OR* [95%CI]	*OR* [95%CI]	*OR* [95%CI]
Non‐MCR (reference)	1	1	1	1
MCR	1.667 [1.223, 2.273]^***^	1.507 [1.100, 2.065]^*^	1.514 [1.105, 2.074]^*^	1.416 [1.023, 1.959]^*^

Abbreviations: CI, confidence interval; MCR, motoric cognitive risk syndrome; non‐MCR, participants without subjective cognitive complaint or slow gait; OR, odds ratios.

^a^ Univariate logistic regression analysis.

^b^ Adjusted for age, gender, level of education, BMI, place of living, and cohabitation status.

^c^ Adjusted for all covariates in model 2 and alcohol consumption, and smoking.

^d^ Adjusted for all covariates in model 3 and emotional and psychiatric problems, hypertension, diabetes, chronic lung disease, arthritis, heart disease, and falls history.

*p < .05, ***p < .001.

### 3.3 MCR and SCC, but not slow gait, associated with falls during follow‐up

Slow gait was not associated with future falls after controlling the confounders, whereas MCR (OR = 1.519, 95%CI = 1.086–2.126) and SCC (OR = 1.241, 95%CI = 1.018–1.513) were highly associated with future falls during follow‐up in the fully adjusted models (Table 3; All variables used in the models displayed in eTable S2).

**TABLE 3:** Logistic regression analysis of future falls (dependent variable) and MCR, SCC, slow gait (independent variables) (*n* = 3748).

variable	Model 1^a^	Model 2^b^	Model 3^c^	Model 4^d^
	*OR* [95%CI]	*OR* [95%CI]	*OR* [95%CI]	*OR* [95%CI]
Healthy (reference)	1	1	1	1
Slow gait	1.005 [0.737, 1.371]	0.979 [0.714, 1.341]	0.981 [0.716, 1.344]	0.883 [0.638, 1.221]
SCC	1.601 [1.328, 1.929]^***^	1.380 [1.138, 1.673]^**^	1.373 [1.132, 1.666]^**^	1.241 [1.018, 1.513]^*^
MCR	1.939 [1.408, 2.669]^***^	1.694 [1.223, 2.346]^**^	1.699 [1.227, 2.353]^**^	1.519 [1.086, 2.126]^*^

Abbreviations: CI, confidence interval; MCR, motoric cognitive risk syndrome; non‐MCR, participants without SCC or slow gait; OR, odds ratios; SCC, subjective cognitive complaint.

^a^ Univariate logistic regression analysis.

^b^ Adjusted for age, gender, level of education, BMI, place of living, and cohabitation status.

^c^ Adjusted for all covariates in model 2 and alcohol consumption, and smoking.

^d^ Adjusted for all covariates in model 3 and emotional and psychiatric problems, hypertension, diabetes, chronic lung disease, arthritis, heart disease, and falls history.

*p < .05, **p < .01, ***p < .001.

### 3.4 Sensitivity analysis

We conducted a sensitivity analysis to explore the robustness of the association between MCR and future falls. Fall history is a well‐established risk factor for falls and is commonly used as a covariate in falls research. By excluding falls history from the analysis, we could explore whether the association between MCR and future falls remained significant even when falls history, which was subject to potential recall bias, was not accounted for. The univariate logistic regression model showed that MCR increased the risk of future falls by 51.5% compared to non‐MCR (eTable S3).

## 4 DISCUSSION

This study examined the associations between MCR and its components (SCC and slow gait) with future falls. The main finding of our study was that MCR and one of its components, SCC, separately predict falls during 3 years of follow‐up, even after adjusting for demographic characteristics, behavioral habits, falls history, and chronic diseases. In contrast, we found no association between slow gait and an increased risk of future falls among the 3478 Chinese elderly aged 60 years and over who participated in the CHARLS. Our study revealed that the risk of future falls is highest among participants with MCR, followed by SCC. These findings underscore the significance of measuring MCR to predict falls.

The prevalence of MCR among community‐dwelling older adults in this study was 5.92%, which was lower than the prevalence in Europe, USA, and Japan (Maggio & Lauretani, 2019). The prevalence of MCR varied across different countries and races due, in part, to different assessment and grading methods of SCC. In a few studies of CHARLS, respondents who rated their memory as “fair” were considered to have SCC (Xu et al., 2021). However, we believe that in the Chinese culture of humility, a memory status that is considered “fair” is acceptable. Therefore, we suggest that only an answer of “poor” to memory should be considered as SCC in our study. Generally, the prevalence of MCR in our study was significantly higher in females (61.71% vs. 38.29%, *p* = .001), those with an education level of primary school or below (91.89% vs. 6.76% vs. 1.35%, *p* < .001), and those living outside the city (89.64% vs. 10.36%, *p* < .001).

In this prospective study, it was found that MCR was more sensitive than non‐MCR in predicting the risk of future falls, thus confirming the results of previous research (Beauchet et al., 2019; Yuan et al., 2021). A systematic review indicated that individuals with MCR had a lower volume of gray matter in the premotor cortex and prefrontal cortex (Bortone et al., 2021a), indicating neurodegeneration in the cortex of MCR. Memory complaint resulted in executive dysfunction (Steinberg et al., 2013), causing a decrease in central processing capacity and an increase in reaction time. Moreover, Sekhon et al.’s (2019) study found that MCR was associated with decreased physical function, including muscle weakness and increased exhaustion. Falls typically occur due to various factors, such as reduced motor and cognitive function, or a cluttered and unfamiliar environmental. The pathological mechanism behind falls in individuals with MCR, where memory complaint and slower gait coexist, is mainly reflected in cognitive motor interference (CMI). CMI refers to the difficulty that older adults may experience when attempting to simultaneously perform a cognitive task and a motor task (McIsaac et al., 2018), such as dealing with impending falls. Thus, when the body reaches the limit of balance, individuals with MCR will present with CMI problems and an inability to immediately control balance due to executive dysfunction and decreased muscle strength. The interference between cognitive and motor function may explain the association between MCR and increased falls risk.

We further explored whether the components of the MCR predicted future falls. The findings of our study showed that SCC, but not slow gait, increased the risk of future falls, which was inconsistent with some existing studies. Specifically, the study found that there was an increased risk of future falls in association with SCC, which differed from the findings of Beauchet et al. (2019), who reported no association of falls with SCC. Slow gait is one predominant domain of gait characteristics that can predict the risk of falls in patients with dementia or MCI (MacAulay et al., 2021), but for the community‐dwelling elderly without MCI in our study, slow gait did not associate with future falls risk during the following 3 years, which was in line with the findings reported by Beauchet et al. (2019). The discrepancy in these findings might be correlated with the temporal relationship between SCC and slow gait (Morris et al., 2016; Nadkarni et al., 2009). Buracchio et al. (2010) reported that slow gait occurred 12 years before cognitive decline in the elderly, which was also verified by Doi et al. (2019), suggesting that slow gait was significantly associated with incident dementia in the full sample. In summary, cognitive performance at baseline might not associate with changes in gait speed during follow‐up, while slow gait could predict a tendency towards worse performance in memory (Bortone et al., 2021b; Doi et al., 2015; Hsu et al., 2017; Mielke et al., 2013; Savica et al., 2017). Therefore, we inferred that SCC could be ahead of slow gait in predicting the risk of future falls during short‐term follow‐up, which might explain why the findings of our study differed from those of existing cross‐sectional studies.

To the best of our knowledge, this is the first prospective study that investigates MCR and its components as predictors of falls in community‐dwelling Chinese elderly based on a large, nationwide sample. Our findings suggest that the influence factors of falls are more likely related to neurocognitive impairment, since MCR and SSC shared memory problems. Despite these findings, several limitations in our study should be acknowledged. First, measuring falls based on recalling may not be entirely accurate due to recall bias. However, SCC may not significantly affect the recall correction rate. In a comparison study of subjective memory complaints and objective memory performance, it was found that there was no significant difference in the ability to recall remote memory, numeric memory, everyday memory, and spatial memory, except for semantic memory (Fyock & Hampstead, 2015). Falls recall is a type of episodic memory that includes remote memory, numeric memory, and spatial memory. Therefore, the self‐reported results of falls in this cohort study could be considered similar to those obtained through objective assessment of falls. Additionally, a previous study showed that healthy older individuals with subjective memory complaints were associated with executive dysfunction, but not impaired delayed recall (Steinberg et al., 2013). This suggests that memory complaints may not significantly affect the correction rate of falls recall. Second, the assessment methods for memory and slow gait need standardization to determine the prevalence and effects of MCR accurately. Our study utilized a self‐rated memory assessment, while others used the memory‐related items from varying scales, such as the three‐item recall test, Mini‐Mental State Examination, and Geriatric Depression Scale (Lord et al., 2020; Shim et al., 2020; Wang et al., 2016). Similarly, slow gait was defined by different classification methods. Our study used the mean and standard deviation of gait speed by groups of age and sex, while others used the default of slow gait speed (Beauchet et al., 2019). Third, the follow‐up period was not long enough to observe the association between baseline slow gait and future falls. For participants who had a decline in gait speed, it might take longer to detect the effect of gait on the future fall risk.

## 5 CONCLUSION

Our prospective cohort study found that the risk of falls is higher in individuals with MCR and SCC, but no association was observed between slow gait and falls. Overall, the study suggests that MCR could be a pragmatic clinical tool for screening fall risk, given its high validity and sensitivity in predicting falls.

## AUTHOR CONTRIBUTIONS

**Wei‐wei Lu**: conceptualization (lead); data curation (lead); methodology (lead); writing—original draft (lead). **Bang‐zhong Liu**: conceptualization (lead); methodology (lead); writing—review and editing (lead); supervision (lead). **Min‐zhi Lv**: data curation (lead); methodology (lead). **Jin‐kang Tu**: data curation (supporting); supervision (supporting). **Zi‐rui Kang**: formal analysis (supporting); supervision (supporting). **Rui‐ping Hu**: data curation (lead); writing—review and editing (lead); conceptualization (lead). **Yu‐lian Zhu**: data curation (lead); writing—review and editing (lead). **Jian Zhang**: conceptualization (lead); supervision (lead); funding acquisition (lead); writing—review and editing (lead).

## CONFLICT OF INTEREST STATEMENT

The authors declare no conflict of interest.

### PEER REVIEW

The peer review history for this article is available at https://publons.com/publon/10.1002/brb3.3044.

## Supporting information


**eTABLE S1** Logistic regression analysis of future falls (dependent variable) and MCR (independent variables) (*n* = 3748).
**eTABLE S2** Logistic regression analysis of future falls (dependent variable) and MCR, SCC, slow gait (independent variables) (*n* = 3748).
**eTABLE S3** Sensitivity analysis.

## Data Availability

The data of this study are available from the corresponding author upon reasonable request.
