# Supplementary material for: Association between motoric cognitive risk syndrome and future falls among Chinese community‐dwelling elderly: A nationwide cohort study
Source: Brain Behav. 2023 May 18;13(7):e3044. doi: 10.1002/brb3.3044 (PMC10338852; doi:10.1002/brb3.3044)
Supplement: Supplementary file 1 — eTABLE S1 Logistic regression analysis of future falls (dependent variable) and MCR (independent variables) (n = 3748). eTABLE S2 Logistic regression analysis of future falls (dependent variable) and MCR, SCC, slow gait (independent variables) (n = 3748). eTABLE S3 Sensitivity analysis. [file BRB3-13-e3044-s001.doc]

**Supplementary data**

**Content**

**eTABLE 1** Logistic regression analysis of future falls (dependent variable) and MCR (independent variables) (n=3748) ....................................................................... P2

**eTABLE 2** Logistic regression analysis of future falls (dependent variable) and MCR, SCC, slow gait (independent variables) (n=3748) ............................................................ P3

**eTABLE 3** Sensitivity analysis ........................................................................ P4

**eTABLE 1** Logistic regression analysis of future falls (dependent variable) and MCR (independent variables) (n=3748)

| variable | Model 1^a^ | Model 2^b^ | Model 3^c^ | Model 4^d^ |
| --- | --- | --- | --- | --- |
|  | *OR* [95%*CI*] | *OR* [95%*CI*] | *OR* [95%*CI*] | *OR* [95%*CI*] |
| non-MCR  (reference) | 1 | 1 | 1 | 1 |
| MCR | 1.667 [1.223, 2.273]^***^ | 1.507[1.100,2.065] | 1.514[1.105, 2.074] | 1.416[1.023, 1.959] |
| Age | - | 1.026[1.011,1.041]^***^ | 1.026[1.011, 1.041]^***^ | 1.027[1.011, 1.042]^***^ |
| female | - | 1.906[1.594,2.279]^***^ | 1.917[1.543, 2.382]^***^ | 1.674[1.335, 2.099]^***^ |
| BMI | - | 0.987[0.963,1.011] | 0.986[0.962, 1.010] | 0.982[0.956, 1.008] |
| Education | - | 1.099[0.913,1.322] | 1.106[0.919, 1.332] | 1.090[0.902, 1.318] |
| non- Cohabitation | - | 1.122[0.896,1.405] | 1.132[0.904, 1.418] | 1.098[0.872, 1.382] |
| Out of city zone | - | 1.218[0.981,1.512] | 1.223[0.985, 1.519] | 1.199[0.959, 1.498] |
| Current drinkers | - | - | 1.195[0.977, 1.461] | 1.152[0.936, 1.417] |
| Current smokers | - | - | 0.859[0.679, 1.088] | 0.855[0.669, 1.092] |
| EPP | - | - | - | 2.103[1.072, 4.124]^*^ |
| Diabetes | - | - | - | 1.278[0.954, 1.713] |
| Hypertension | - | - | - | 0.985[0.810, 1.197] |
| Heart disease | - | - | - | 1.057[0.822, 1.358] |
| CLD | - | - | - | 0.951[0.733, 1.235] |
| Arthritis | - | - | - | 1.161[0.973, 1.384] |
| Falls history | - | - | - | 2.731[2.287, 3.261] |

Abbreviations: MCR, motoric cognitive risk syndrome; non-MCR, participants without subjective cognitive complaints or slow gait; OR, odds ratios; CI, confidence interval; EPP, Emotional and Psychiatric Problems; CLD, Chronic Lung Disease.

^a^ univariate logistic regression analysis; ^b^ adjusted for age, gender, level of education, BMI, place of living, and cohabitation status; ^c^ adjusted for all covariates in model 2 and alcohol consumption, and smoking; ^d^ adjusted for all covariates in model 3 and emotional and psychiatric problems, hypertension, diabetes, chronic lung disease, arthritis, heart disease, and falls history. **p*<0.05, ***p*<0.01, ****p*<0.001.

-, means no value.

**eTABLE 2** Logistic regression analysis of future falls (dependent variable) and MCR, SCC, slow gait (independent variables) (n=3748)

| variable | Model 1^a^ | Model 2^b^ | Model 3^c^ | Model 4^d^ |
| --- | --- | --- | --- | --- |
|  | *OR* [95%CI] | *OR* [95%CI] | *OR* [95%CI] | *OR* [95%CI] |
| Healthy(reference) | 1 | 1 | 1 | 1 |
| Slow gait | 1.005 [0.737, 1.371] | 0.979[0.714, 1.341] | 0.981[0.716, 1.344] | 0.883[0.638, 1.221] |
| SCC | 1.601 [1.328, 1.929]*** | 1.380 [1.138, 1.673]** | 1.373 [1.132, 1.666]** | 1.241 [1.018, 1.513]* |
| MCR | 1.939 [1.408, 2.669]*** | 1.694 [1.223, 2.346]** | 1.699 [1.227, 2.353]** | 1.519 [1.086, 2.126]* |
| Age | - | 1.026[1.011, 1.040]*** | 1.026[1.011, 1.040]*** | 1.026[1.011, 1.042]*** |
| female | - | 1.826[1.525, 2.187]*** | 1.844[1.482, 2.294]*** | 1.626[1.295, 2.041]*** |
| BMI | - | 0.988[0.964, 1.012] | 0.987[0.962, 1.012] | 0.983[0.957, 1.010] |
| Education | - | 1.067[0.886, 1.285] | 1.074[0.891, 1.294] | 1.073[0.887, 1.299] |
| non-Cohabitation | - | 1.130[0.902, 1.416] | 1.140[0.910, 1.429] | 1.107[0.879, 1.395] |
| Out of city zone | - | 1.180[0.949, 1.467] | 1.186[0.953, 1.474] | 1.171[0.936, 1.465] |
| Current drinkers | - | - | 1.190[0.973, 1.454] | 1.146[0.931, 1.410] |
| Current smokers | - | - | 0.869[0.686, 1.101] | 0.860[0.673, 1.099] |
| EPP | - | - | - | 2.095[1.074, 4.087]* |
| Diabetes | - | - | - | 1.272[0.950, 1.705] |
| Hypertension | - | - | - | 0.976[0.802, 1.186] |
| Heart disease | - | - | - | 1.057[0.822, 1.358] |
| CLD | - | - | - | 0.958[0.739, 1.244] |
| Arthritis | - | - | - | 1.142[0.957, 1.363] |
| Falls history | - | - | - | 2.706[2.264, 3.233] |

Abbreviations: MCR, motoric cognitive risk syndrome; SCC, subjective cognitive complaints; OR, odds ratios; CI, confidence interval; EPP, Emotional and Psychiatric Problems; CLD, Chronic Lung Disease.

^a^ univariate logistic regression analysis; ^b^ adjusted for age, gender, level of education, BMI, place of living, and cohabitation status; ^c^ adjusted for all covariates in model 2 and alcohol consumption, and smoking; ^d^ adjusted for all covariates in model 3 and emotional and psychiatric problems, hypertension, diabetes, chronic lung disease, arthritis, heart disease, and falls history. **p*<0.05, ***p*<0.01, ****p*<0.001.

-, means no value.

Sensitivity analysis: using univariate logistic regression model after additionally excluding the participants with fall history (eTable 3).

**eTABLE 3** logistic regression analysis of future fall (dependent variable) and MCR (independent variables) (n=2798)

| variable | *OR* | *SE* | *p* | 95%*CI* |
| --- | --- | --- | --- | --- |
| MCR | 1.516 | 0.319 | 0.048 | 1.003, 2.291 |

Abbreviations: MCR, motoric cognitive risk syndrome; OR, odds ratios; SE, standard error; CI, confidence interval.
